# Take Action or Worry? Older Adults’ Anticipated Responses to Concerning Cognitive Assessment Results

**DOI:** 10.1093/geroni/igaa057.1179

**Published:** 2020-12-16

**Authors:** Claudia Jacova, Sara Wong, Samantha Smith

**Affiliations:** 1 Pacific University, Hillsboro, Oregon, United States; 2 Student, Hillsboro, Oregon, United States

## Abstract

Under a third of older adults (28%) report having ever received an assessment for cognitive problems in US primary care settings. Patient resistance is cited as a major reason cognitive assessments are not performed. Theoretical models emphasize the role of anticipated benefits and harms in shaping health behaviors. Accordingly, here we investigated older adults’ anticipated actions and worries regarding their cognitive assessment results. A total of 393 community-dwelling respondents between ages 50 and 91, 65% female, 89% college/university-educated, with no diagnosed cognitive disorder, completed Attitudes Around Cognitive Testing (AACT) at primary care sites (n=98) and through an online platform (www.mturk.com) (n=298). AACT examines older adults’ preferences and concerns about cognitive assessment. It includes questions about actions participants would take and worries they would have if assessment results indicated cognitive problems. Willingness to take part in testing (yes or unsure/no) was also assessed. We found that seeking a formal diagnosis (84%), talking to family about healthcare (77%), and planning one’s own future (70%) were highly endorsed actions, and becoming depressed (48%), becoming anxious (47%), and losing driving privileges (41%) highly endorsed worries. Logistic regression showed that total worries and worry-action difference scores predicted reduced willingness (OR=0.84, CI=0.75-0.93 and OR=0.82, CI=0.74-0.82, respectively), whereas total actions did not. Our results suggest that older adults view concerning cognitive assessment outcomes as an opportunity for taking action as well as a reason for worrying. Both worries and actions appear to play a role in deciding whether to take part in a cognitive assessment.

